# Snoring and aortic dimension in Marfan syndrome

**DOI:** 10.1007/s41105-022-00413-5

**Published:** 2022-08-11

**Authors:** Mudiaga Sowho, Mariah Potocki, Frank Sgambati, Enid Neptune

**Affiliations:** 1 Division of Pulmonary and Critical Care Medicine, Johns Hopkins School of Medicine, Baltimore, MD USA; 2 Center for Interdisciplinary Sleep Research and Education, Johns Hopkins School of Medicine, Baltimore, MD USA

**Keywords:** Snoring, OSA, Aortic diameter, Marfan syndrome

## Abstract

Recent reports suggest that self-reported snoring, which is a feature of obstructive sleep apnea, is associated with aortic enlargement in Marfan syndrome (MFS). Objective assessment of snoring although lacking, could provide a rational for OSA screening in MFS patients. Our goal in this study was to examine the association between objective measurements of snoring with OSA and aortic size in persons with MFS. Consecutive persons with MFS who reported snoring were recruited at Johns Hopkins, completed the Epworth Sleepiness Scale (ESS) and underwent overnight polysomnography during which inspiratory sound was captured. We measured breath-by-breath peak decibel levels and snoring was defined as flow limitation with sound ≥ 40 dB(A). OSA was defined as an apnea–hypopnea-index (AHI) ≥ 15 or AHI: 5–15 and ESS > 10. Participants’ aortic data were collated to ascertain aortic root diameter. Regression models were used to determine the relationship of snoring breath% with OSA and aortic root diameter. In our cohort (M|F:13|16, Age: 37.0 ± 15.5 years, Aortic diameter; 38.9 ± 4.8 mm), a 1-unit increase in snoring breath percentage increased the odds of having OSA by 5% in both the unadjusted (OR = 1.05, *p* = 0.040) model, and a model adjusted for age and sex (OR = 1.05, *p* = 0.048). Similarly, a 10-unit increase in snoring breath percentage was associated with a 1 mm increase in contemporaneous aortic-root-diameter in both unadjusted (*β* = 0.09, *p* = 0.007), and adjusted (*β* = 0.08, *p* = 0.023) models. Objective snoring assessment could provide a means for identifying persons with MFS who need sleep studies, who may also be at risk for more severe aortic disease.

## Introduction

Snoring and associated obstructive sleep apnea (OSA) are highly prevalent in persons with Marfan syndrome (MFS) [1–3], a genetic disorder that compromises connective tissue leading to thoracic aortic aneurysms and eventual dissection or rupture [4, 5]. We recently reported that accumulated nocturnal exposure to snoring increases cardiovascular stress in MFS persons [6].

Our survey of Johns Hopkins MFS patients using the STOP-BANG sleep apnea questionnaire revealed that those who reported loud snoring had larger aortic root diameters. The aorta dilatation rate was greater in younger subjects who reported loud snoring compared to those who did not report loud snoring [7]. Thus, snoring may have important consequences for aortic health in MFS, and if assessed objectively may serve as an indicator of underlying OSA as well as a marker for aortic enlargement.

In the current analyses, our goal was to quantify overnight snoring and examine its association with OSA and contemporaneous aortic root diameter in a group of self-reported snorers with MFS. We hypothesized that in MFS persons, objective measures of snoring will (a) predict OSA and (b) be positively associated with aortic root diameter.

## Methods

Thirty consecutive MFS patients who reported habitual snoring were recruited from the Johns Hopkins Vascular Connective Tissue Disorders Clinic and the Marfan Foundation. Participants completed a packet of sleep disordered breathing questionnaires including the STOP-BANG, Berlin questionnaire and the Epworth sleepiness scale (ESS) [1, 8, 9], and also provided contemporaneous aortic information verified with their medical records. All participants underwent overnight in-laboratory polysomnography, but one recording was excluded because participant’s sleep time was less than 2 h, thus data were not adequate for our analyses. Sleep staging and respiratory analyses were done using the American Academy of Sleep Medicine (AASM) criteria [10] and OSA was defined as an AHI ≥ 15 events/h or an AHI between 5 and 15 events/h with an ESS score > 10 [11, 12]. The study was approved by the Johns Hopkins Institutional Review Board.

### Measurement and analyses of snoring

**Fig. 1:**
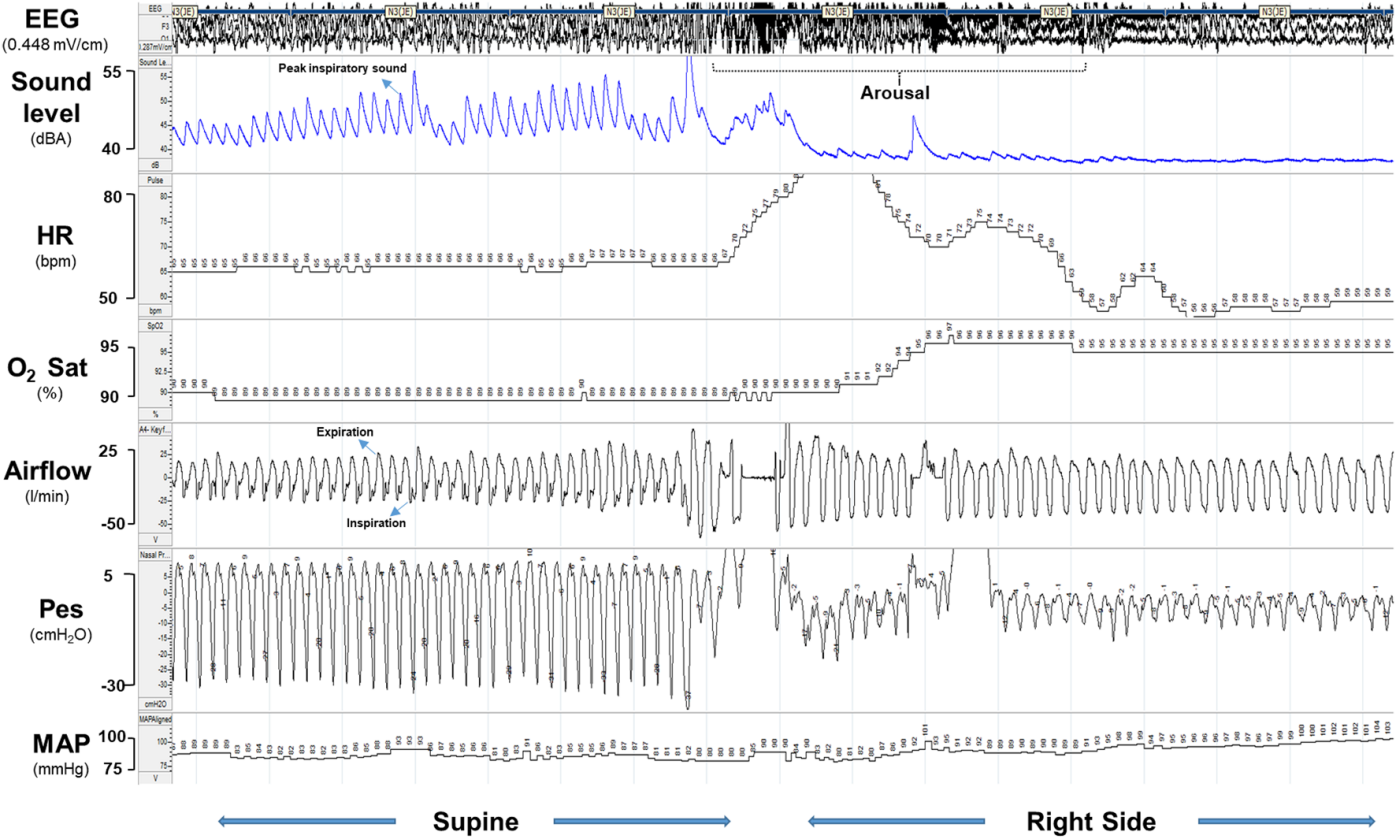
Electro-encephalogram (EEG), sound level, oxygen saturation, airflow, heart rate (HR), esophageal pressure (Pes) and mean arterial pressure (MAP) channels**,** during a 3-min sleep period

Snoring was captured with a high-accuracy class 2 digital sound pressure level meter with an accuracy ± 1.4 dB (DT-8851, Ruby Electronics, Saratoga, CA) in adherence with IEC 61672–1 standard. The sound pressure level meter was affixed 65 cm above the head position of the bed during the study night to approximate the distance between the head of the bed partner and snorer. We defined snoring as a peak sound ≥ 40 dB(A) [13], during inspiration (Fig. 1) and tallied the number of breaths with peak sound ≥ 40 dB(A) to estimate the proportion of snoring breaths over the entire sleep period, which we termed the snoring breath %.

### Statistical analyses

Logistic regression was used to model the association between snoring breath % as a continuous predictor variable and the presence of OSA as a dichotomous outcome variable. Linear regression was used to model the association between snoring breath % as a continuous predictor variable and aortic root diameter as a continuous outcome variable. Associations were examined in both unadjusted models and models adjusted for age and sex. Analyses were performed using R. Two-tailed *p* values of less than 0.05 were considered to indicate statistical significance.

## Results

### Participant characteristics

Twenty-nine self-reported snorers with MFS underwent overnight polysomnography with over 4 h of sound monitoring during sleep. The group had a mean age of 37.0 ± 15.5 years, BMI of 26.1 ± 4.8 kg/m^2^, and 55% were women. The median snoring breath % was 23.5 (IQR:4.0–38.5) and 14 participants had OSA. Among those with native aortas, the mean aortic root diameter was 3.9 ± 0.5. Eighty-six percent of subjects were on a beta-blocker, an angiotensin receptor blocker (ARB) or both (Table 1).

**Table 1:** Participant Characteristics

	*N* = 29
Clinical characteristics	
Sex, w:m	16:13
Age, years	37.0 ± 15.5
Height, cm	183.6 ± 11.1
Body mass index, kg/m^2^	26.1 ± 4.8
Snoring breath, %	23.5 (4.0–38.5)
OSA, *n* (%)	14 (48)
Aortic root diameter, mm	38.9 ± 4.7
Aortic root replacement, *n* (%)	7 (24)
Blood pressure medications	
Beta-blocker only, *n* (%)	3 (10)
ARB only, *n* (%)	12 (41)
Beta-blocker and ARB, *n* (%)	10 (35)
None, *n* (%)	4 (14)

Data are reported as means ± standard deviations, median and interquartile range, and absolute values with frequency, *n* (%)

*OSA* obstructive sleep apnea, *ARB* angiotensin receptor blocker

### Association of snoring breath % with OSA and aortic root diameter

**Fig. 2:**
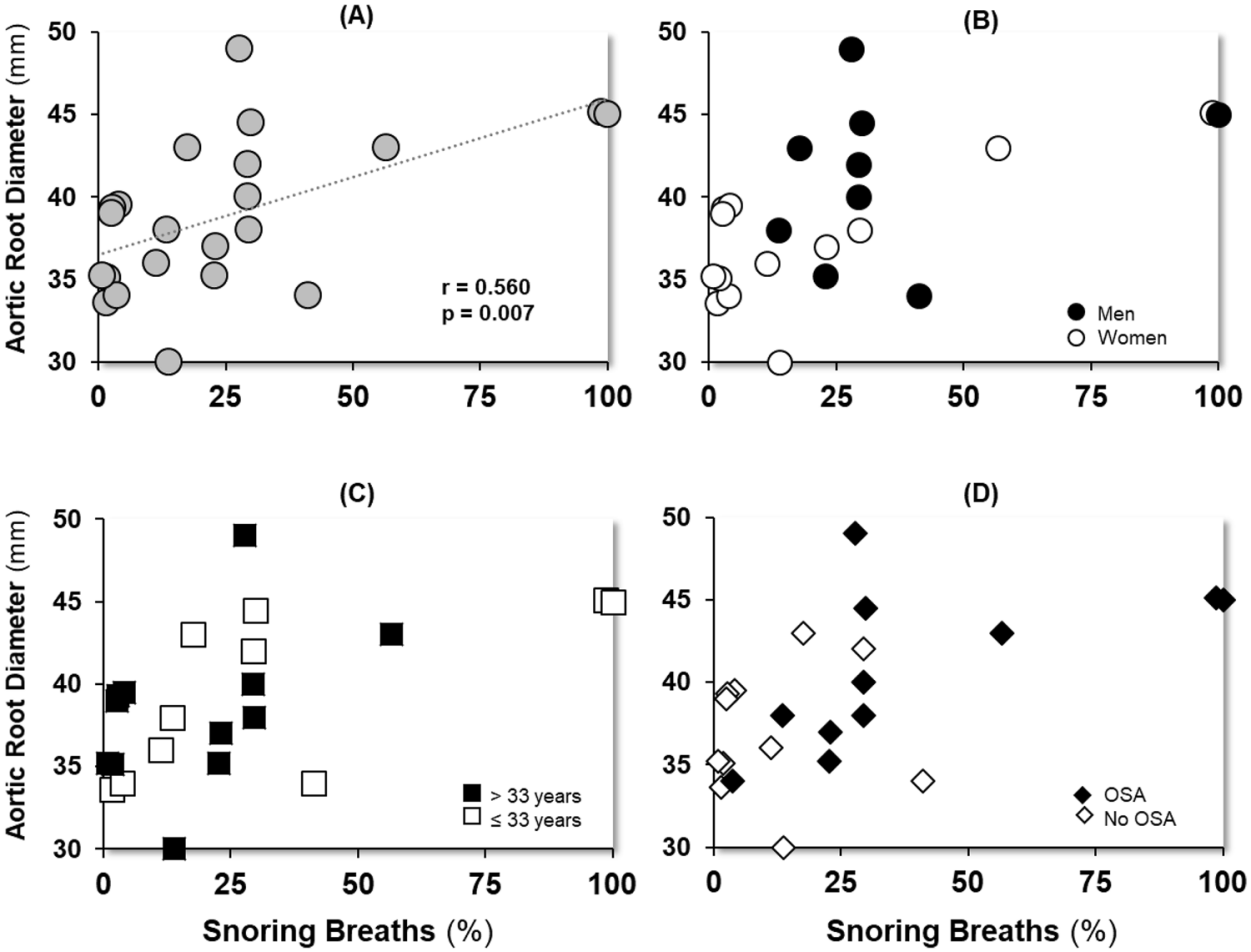
Association of snoring breath percent and aortic diameter. Panel (**A**), all participants (*r* = 0.560, *p* = 0.007), Panel (**B**), men (*r* = 0.242, *p* = 0.531) and women (*r* = 0.712, *p* = 0.006); Panel (**C**), participants below (*r* = 0.668, *p* = 0.035) and above (*r* = 0.434, *p* = 0.159) the median age, and Panel (**D**), participants with (*r* = 0.597, *p* = 0.053) and without (*r* = 0.069, *p* = 0.841) OSA

Regression analyses revealed that a higher snoring breath % increased the likelihood of having OSA, such that a 1-unit increase in snoring breaths was associated with 5% higher odds of having OSA in the unadjusted (OR = 1.05, 95% CI = 1.01–1.10, *p* = 0.040), and adjusted (OR = 1.05, CI = 1.01–1.10, *p* = 0.048) models (Table 2). Similarly, we found a positive association between snoring breath % and aortic root diameter (Fig. 2), such that a 10-unit increase in snoring breath percentage was associated with a 1 mm increase in aortic root diameter in both unadjusted (*β* = 0.09, *p* = 0.007), and adjusted models (*β* = 0.08, *p* = 0.023) (Table 2).

**Table 2:** Association of Snoring breaths % with OSA and Aortic root diameter

	OSA			
	OR	CI	*P*	N
Unadjusted				
Snoring breath %	1.05	1.01–1.10	**0.040**	29
Adjusted				
Snoring breath %	1.05	1.01–1.10	**0.048**	29
Male sex	2.09	0.34–13.4	0.416	29
Age	1.03	0.97–1.10	0.930	29

	Aortic root diameter			
	*β*	SE	*P*	*N*
Unadjusted				
Snoring breath %	0.09	0.031	**0.007**	22
Adjusted				
Snoring breath %	0.08	0.033	**0.023**	22
Male sex	2.67	1.802	0.156	22
Age	0.01	0.054	0.930	22

Statistically significant *P* values are in bold (*P* < 0.05)

*OSA* obstructive sleep apnea, *OR* odds ratio, *CI* confidence interval, *β* beta coefficient, *SE* standard error

## Discussion

Our cross-sectional analyses in self-reported snorers with MFS revealed a positive relationship between the proportion of snoring breaths during sleep and (a) OSA, and (b) aortic size even after adjusting for age and sex.

A previous study demonstrated that the proportion of snoring breaths during sleep was a predictor of OSA in the general population [13]. Our study confirms this finding in persons with MFS, a genetic disorder characterized by aortic pathology [4, 14, 15] and sleep disordered breathing [1–3]. Our results further reveal that a 1-unit increase in snoring breaths percentage confers a 5% higher likelihood of OSA, and that a 10-unit increase in snoring breaths percentage associates with a 1 mm increase in aortic root diameter. The mean rate of aortic dilatation is 2.6 mm/year MFS patients [16, 17] compared with a 1.5 mm/year in the general population [18, 19]. Given the susceptibility for aortic morbidity in MFS, the additional 1 mm dilation per year is likely a contributor to increased aortic adverse events. Our data suggest that an increase in snoring breath percentage by 10 units can confer 1 mm growth in aortic size, potentially increasing the risk for aortic dissection and rupture in the MFS population, who already have larger aortic diameters (Table 2) relative to the general population [19, 20]. Although adverse aortic health consequences are prevalent in MFS, our findings imply that snoring is a possible contributor or a phenotype that represents greater aortic disease severity.

Our findings may be explained by the associated pathophysiologic changes that occur with snoring, which may increase aortic stress [6]. As shown in Fig. 1, snoring resulted in large swings in pleural pressure, decrease in oxygen saturation and an increase in heart rate. Since persons who snore are likely to also have OSA, fluctuations in blood pressure caused by apneas and hypopneas [21, 22] will further compound the cardiovascular stress due to snoring [6].

Our findings indicate that objective measures of snoring constitute a strong predictor for concomitant OSA in MFS. Therefore, increased availability of home-based assessments of snoring can facilitate OSA screening in the MFS community and offer a rationale for obtaining formal sleep studies. Our findings also support the development of strategies to elucidate dose–response relationships between snoring and markers of contemporaneous aortic stress, as well as long-term adverse aortic events in MFS. Further studies are still needed to validate our findings in larger cohorts and to determine whether treatment of snoring with or without OSA mitigates aortic disease progression in MFS.

## Data Availability

The datasets generated during and/or analyzed during the current study are not publicly available because it contains information that may compromise research participant privacy/consent, but are available from the corresponding author (MS) on reasonable request.
